# Factors regulating growth pattern and condition factor of an amphibious fish *Periophthalmus gracilis* living in the Mekong Delta

**DOI:** 10.7717/peerj.13060

**Published:** 2022-03-04

**Authors:** Quang Minh Dinh, Ton Huu Duc Nguyen, Ngon Trong Truong, Lam Nguyen-Ngoc

**Affiliations:** 1 Department of Biology, School of Education, Can Tho University, Can Tho, Vietnam; 2 Department of Molecular Biotechnology, Biotechnology Research and Development Institute, Can Tho University, Can Tho, Vietnam; 3 Department of Marine Plankton, Institute of Oceanography, Vietnam Academy of Science and Technology, Khanh Hoa, Vietnam

**Keywords:** Isometry, Mudskipper, Negative allometry, Positive allometry, Vietnam

## Abstract

Growth pattern and condition factor (*CF*) are essential to fish resource assessment but limited to *Periophthalmus gracilis*—an amphibious fish living in the mudflats along the Indo-Pacific regions, including the Mekong Delta (MD), Vietnam. This study lasted from April 2020 to March 2021 to verify if their growth pattern and *CF* change with sex, size, season, month and site. The total length and weight of 486 individuals (236 females and 250 males) were 2.9–5.9 cm and 0.13–1.66 g, respectively. The mudskipper displayed negative allometry as the slope value (*b* = 2.69 ± 0.06) obtained from length and weight (*LWR*) was significantly less than 3 (*p* < 0.01), indicating that most fish specimens were caught in the immature stage. The fish growth pattern did not change with sex as both males and females displayed negative allometry but varied by size since the mudskipper showed negative allometry in the immature group and isometry in the mature group. Likewise, growth type changed with season since fish showed negative allometry in the dry season but isometry in the wet season. As the slope value (*b*) varied by site and month, the mudskipper displayed spatiotemporal growth patterns, ranging from negative to positive allometry. The *CF* was impacted by sex as this value of females (1.09 ± 0.02) was higher than that of males (0.96 ± 0.01, *p* < 0.01). Besides, *CF* was regulated by fish length since this value was higher in the mature group (1.12 ± 0.03) than in the immature group (1.01 ± 0.01, *p* < 0.01). Likewise, *CF* was affected by season as this value was higher in the wet season (1.05 ± 0.02) than in the dry season (0.99 ± 0.01, *p* < 0.01). Although the *CF* varied with site and month variables (*p* < 0.01), this value (1.02 ± 0.01) was generally higher than 1, showing fish adapted well to their habitat. The fish length at first capture should be increased to exploit this species sustainably.

## Introduction

Gobies are known as one of the critical dietary components due to their high protein content (Nguyen, 2001), but their stocks are subject to plummeting caused by overexploitation, environmental degradation, and climate changes (Thai et al., 2012). Fisheries management is tackled to economic, social and biological pressure and regulated by fish biometrics (Zargar et al., 2012). Length-weight relationship and well-being condition are essential to fish biometric estimation (Froese, 2006; Mahmood et al., 2012; Truong et al., 2021).

The relationship between fish length and weight (*LWR*) plays an essential role in evaluating the growth and biomass of a fish population (Khaironizam & Norma-Rashid, 2002; Mahmood et al., 2012; Jin et al., 2015; Dinh, 2016a; Lam & Dinh, 2021) and in assessing fisheries management (Froese, 1998; Froese & Pauly, 2000; Gonzalez Acosta, De La Cruz Agüero & De La Cruz Agüero, 2004; Jin et al., 2015; Phan et al., 2021b). In addition, the growth pattern determined from the slope parameter (*b*) of *LWR* (Froese, 2006) and condition factor (*CF*) play a vital role in fish ecological adaptation understanding (Abdoli et al., 2009; Dinh et al., 2016). Fish growth patterns and *CF*, including gobies, are affected by sexual, intraspecific and spatiotemporal variables (Froese, 2006; Abdoli et al., 2009; Lam & Dinh, 2021; Phan et al., 2021b; Truong et al., 2021). However, these data are limited in mudskippers, one of the gobiid fish groups, in the Mekong Delta (MD).

The mudflats and mangroves are habitats of many species of animals, including fishes (Sanders et al., 2010; Sasmito et al., 2020). Mudskipper is a unique fish group that lives mainly in these habitats (Murdy, 1989) and can obtain oxygen directly from the air using their skin and gills (Jaafar et al., 2009). *Periophthalmus gracilis* is one of three species of the *Periophthalmus* in MD (Tran et al., 2013) and 19 species in the world (Murdy & Jaafar, 2017). It occurs quite frequently in mudflat and mangrove regions (Murdy, 1989; Kottelat et al., 1993; Jaafar & Hou, 2012; Tran et al., 2013; Tran et al., 2020; Dinh et al., 2021b; Tran et al., 2021a) and can move flexibly in and out of the water to catch preys (Wicaksono et al., 2020). In MD, *P. gracilis* is being captured for food supply that leads to the reduction of fish resources day by day; however, there is no data on its biology, ecology, and which factors affect the fish population. Therefore, this study was conducted to understand its growth pattern and *CF*, which can be used to realize fish ecological adaptation and fishing status.

## Material and Methods

### Study site and fish analysis

**Figure 1:**
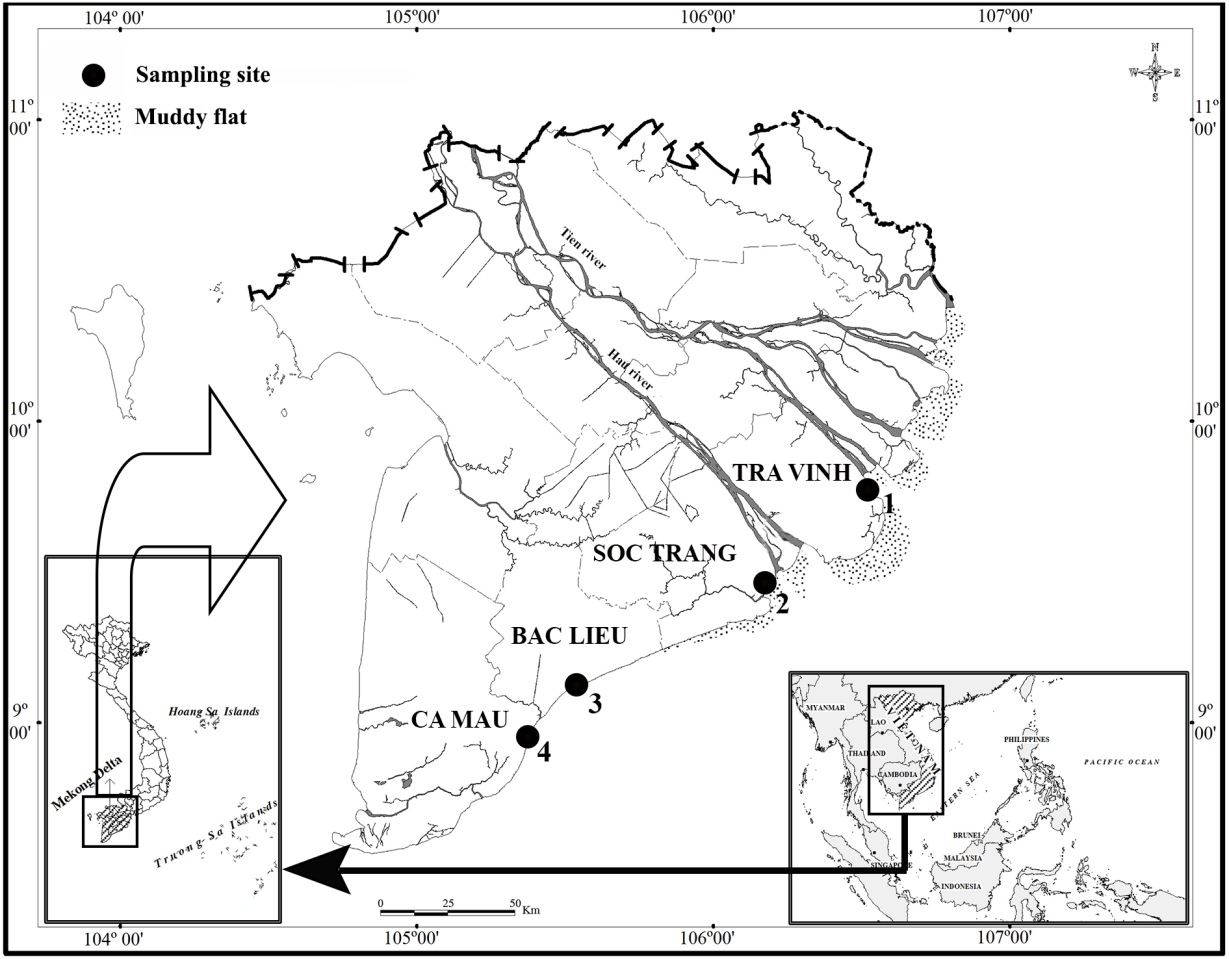
Distribution map of sampling sites. (•: Sampling site; 1: Duyen Hai, Tra Vinh; 2: Tran De, Soc Trang; 3: Dong Hai, Bac Lieu; 4: Dam Doi, Ca Mau) (Dinh, 2018).

This study lasted for 12 months from April 2020 to March 2021 at four sites in the estuarine and coastal regions in MD, including Duyen Hai - Tra Vinh (TV, 9°40′29.5″N 106°34′49.5″E); Tran De - Soc Trang (ST, 9°26′19.7″N 105°10′48.1″E); Dong Hai - Bac Lieu (BL, 9° 05′50.5″N 105°29′54.7″E) and Dam Doi - Ca Mau (CM, 8°58′10.4″N 105°22′58.9″E) (Fig. 1). There are two seasons in these sites, including the dry season from January to May (with no rain) and the wet season from June to December (with heavy rain) (Le et al., 2006). The pH ranged 7.6−8.0, and the salinity varied widely from 12.3 to 23.5‰. The pH change depends on site but not season, whereas the salinity variation gives the opposite result (Dinh et al., 2021a). The primary vegetation at TV and ST is *Sonneratia caseolaris* and *Rhizophora apiculata*, while *R. apiculata* is the predominant vegetation in BM and CM, according to our observation.

An area of 120 m^2^ (6-m width × 20-m length) in each site was chosen to monthly catch fish species at night as fish was so active in the daytime, from April 2020 to March 2021. Fish samples were collected monthly by hand-catching for 4-hours continuously during low tide. Semi-tides represent the site samples, and during low tide, fish gather in large numbers on the mudflats. Fish specimens were easily distinguished from congeners as *P. gracilis* was covered by many irregular blackish dots, whereas *P. chrysospilos* and *P. variabilis* were surrounded by tiny orange spots and greyish brown (Murdy & Jaafar, 2017). MS222 (30 mg/l) dissolved with water taken from the sampling site was used to anaesthetize the fish specimens before being preserved in formalin buffer 5% (dilute from formalin with the ratio of 1 formalin: 9 water taken from the sampling site) and shipped to the laboratory. Fish was sexing using genital papilla, which was triangle males and oval shape in females. Then, fish total length (*TL*) was measured using a ruler to the nearest 0.1 cm, and fish weight (*W*) was weighted using an electric scale to the nearest 0.01 g. The Council for Science and Education, School of Education, Can Tho University approves the fish use in this study (Animal Welfare Assessment number: BQ2020-03/KSP).

### Data analysis

The length-weight relationship of fish was defined as *W=a* ×*TL*^*b*^ (*W*: fish weight, *TL*: fish total length, *a*: intercept parameter and *b*: slope parameter) (Ricker, 1973). The condition factor (*CF*), according to Le Cren (1951), was calculated as *CF=W/* (*a* ×* TL*^*b*^). *T*-test verified if the *b* value obtained from the *LWR* s was ≈3. Fish showed positive allometry (*b* >  3), negative allometry (*b* < 3) and isometry (*b* ≈3) (Martin, 1949). *T*-test qualified whether *CF* was regulated by sex, size and season (dry season and wet season), while one-way ANOVA verified if *CF* changed with month and site (Mahmood et al., 2012). *T*-test confirmed if *CF* was ≈1, whereas General Linear Model qualified gender × season, gender × site and season × site affecting *CF* (Dinh, 2016a). Fish was divided into the immature group when *TL* <*L*_*m*_ and the mature group if *TL* ≥*L*_*m*_. Fish length at first maturity (*L*_*m*_) of males and females at each site was calculated from the formula: *P* = 1/(1 + exp[ − *r* × (*TL* − *L*_*m*_)]) (P: proportion of mature individuals in a length class; *TL*: fish total length; and r: model parameter) (Zar, 1999). *L*_*m*_ was length at first maturity of female and male which was 5.0 cm and 5.7 cm in TV; 4.6 cm and 5.8 cm in ST; 4.9 cm and 5.2 cm in BL; and 6.2 cm and 5.9 cm in CM, respectively (Dinh et al., in press). Before weighting to the nearest 0.01 mg, ovarian and testicular development stages were classified into six developmental stages according to the methods of Dinh et al. (2020). Data analysis was performed using SPSS v.21, and all tests were set at *p* < 0.05. To lessen the Type I error of all tests, the Benjamini–Hochberg procedure was performed (Benjamini & Hochberg, 1995; McDonald, 2014).

## Results

### Growth pattern

**Figure 2:**
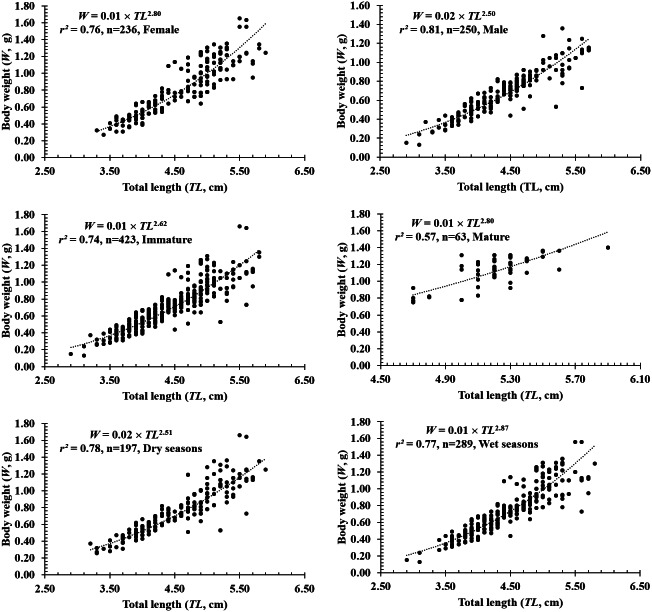
The length-weight relationship of *P. gracilis* in different sexes, sizes and seasons.

**Figure 3:**
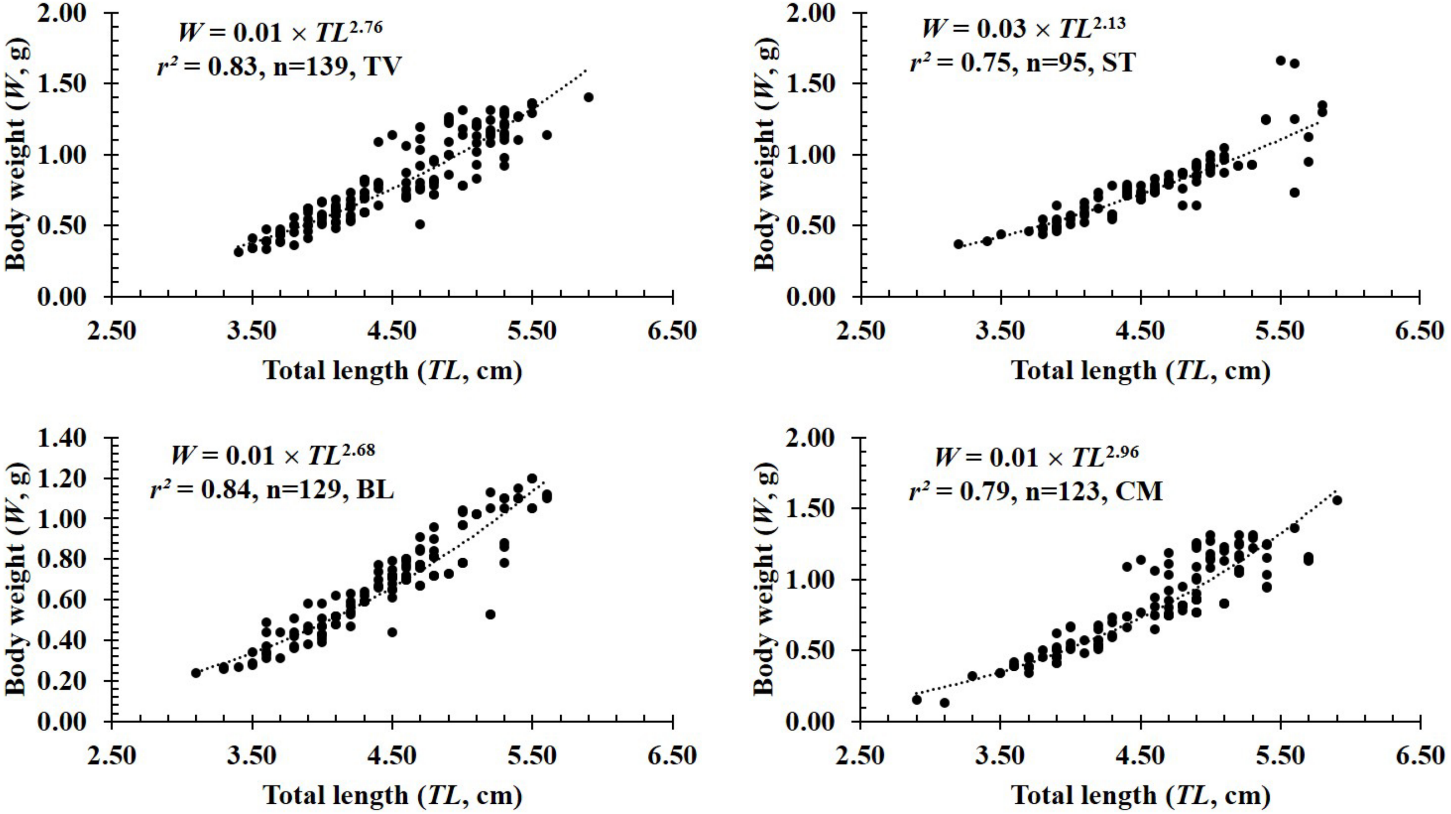
The length-weight relationship of *P. gracilis* in different sites. TV, Duyen Hai–Tra Vinh; ST, Tran De–Soc Trang; BL, Dong Hai–Bac Lieu; CM, Dam Doi–Ca Mau.

**Figure 4:**
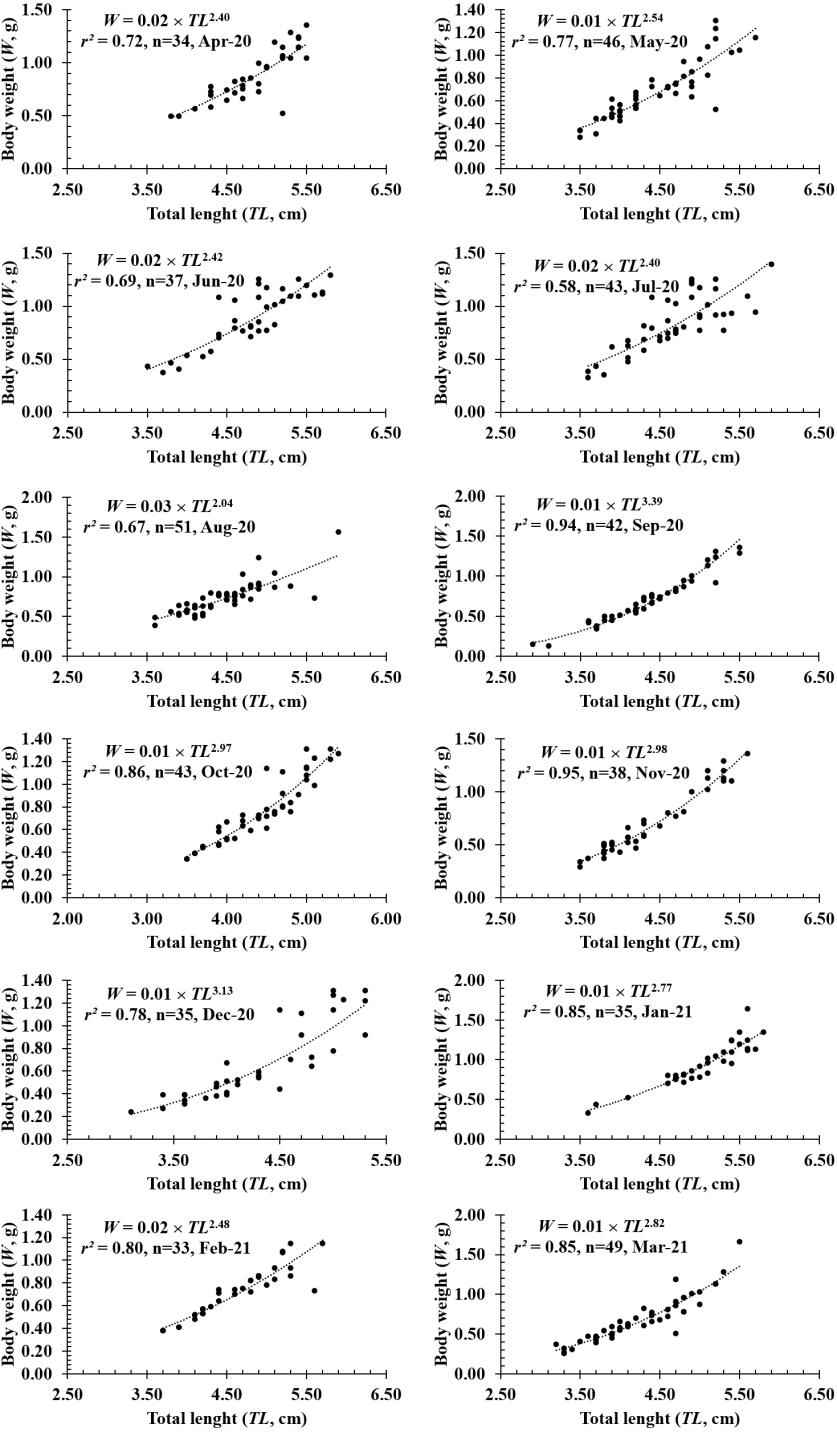
The length-weight relationship of *P. gracilis* in different months.

The total length and weight of 486 individuals collected at four sites from Tra Vinh to Ca Mau were 2.9–5.9 cm and 0.13–1.66 g, respectively (Table 1). The *LWRs* of *P. gracilis* in different fish sexes, sizes, seasons, sites and months were presented in Figs. 2–4. The growth pattern of *P. gracilis* was obtained from the slope value (b) of the length-weight relationship. Specifically, as a slope *b* (2.69 ± 0.06 SE) got from *LWR* was significantly less than the threshold of 3 (*n* = 486, *df* = 484, *p* <  0.01, *t* =  − 5.46), *P. gracilis* belonged to a negative allometric growth pattern.

**Table 1:** Number of samples by sex, site and month.

Months	Duyen Hai -Tra Vinh				Tran De - Soc Trang				Dong Hai - Bac Lieu				Dam Doi - Ca Mau			
	Male	Female	TL range	W range	Male	Female	TL range	W range	Male	Female	TL range	W range	Male	Female	TL range	W range
Apr-20	3	7	3.8–5.5	0.50–1.36	4	3	4.3–5.4	0.78–1.24	7	5	4.5–1.2	0.53–1.15	2	3	4.7–5.4	0.85–1.25
May-20	3	12	3.5–5.2	0.34–1.31	6	3	3.9–4.9	0.49–0.79	8	6	3.5–1.1	0.28–1.05	5	3	4.2–5.7	0.57–1.16
Jun-20	1	8	4.4–5.4	0.87–1.26	3	5	3.5–5.8	0.44–1.30	5	5	4.6–1.2	0.72–1.20	6	4	3.7–5.7	0.38–1.14
Jul-20	5	7	3.6–5.9	0.39–1.40	4	4	4.5–5.7	0.68–0.95	8	4	3.6–1.1	0.33–1.10	3	8	4.4–5.4	0.75–1.26
Aug-20	7	6	3.8–4.4	0.54–0.80	6	4	3.9–5.6	0.64–1.05	11	4	3.6–0.9	0.49–0.90	8	5	3.6–5.9	0.39–1.56
Sep-20	1	11	3.8–5.5	0.50–1.36	5	3	4.5–5.2	0.72–0.94	8	2	3.6–0.9	0.44–0.85	5	7	2.9–5.2	0.13–1.31
Oct-20	5	9	3.6–5.4	0.39–1.31	6	2	4.2–5.1	0.73–0.99	11	1	3.9–1.0	0.47–1.04	3	6	3.5–5.0	0.34–1.15
Nov-20	5	3	4.6–5.4	0.77–1.20	5	3	3.8–4.5	0.44–0.68	7	4	3.5–0.5	0.29–0.53	1	10	3.8–5.6	0.50–1.36
Dec-20	4	4	3.8–5.3	0.36–0.92	3	3	3.4–4.8	0.39–0.64	4	4	3.1–0.4	0.24–0.44	3	10	3.6–5.3	0.39–1.31
Jan-21	6	4	3.6–5.6	0.33–1.35	3	3	5.0–5.8	0.92–1.64	5	3	4.6–1.2	0.77–1.20	7	4	3.7–5.7	0.44–1.24
Feb-21	7	5	3.7–5.3	0.38–1.15	4	1	4.4–5.6	0.71–0.93	4	3	4.1–0.9	0.48–0.86	6	3	3.7–5.7	0.38–1.15
Mar-21	7	9	3.4–5.3	0.31–1.28	9	3	3.2–5.5	0.37–1.66	6	4	3.3–1.1	0.26–1.13	5	6	3.3–4.9	0.32–1.19
Total	54	85	3.4–5.9	0.31–1.40	58	37	3.2–5.8	0.37–1.66	84	45	3.1–5.6	0.24–1.20	54	69	2.9–5.9	0.13–1.56

**Notes.**

TL total length W weight

In terms of sex, although the slope *b* value of females (2.80 ± 0.08 SE, *n* = 236) was higher than that of males (2.50 ± 0.07 SE, *n* = 250), both males and females displayed negative allometry as these values were <3 (*df*_*females*_ = 234, *t*_*females*_ = −2.40, *p*_*females*_ = 0.02; *df*_*males*_ = 248*, t*_*males*_ = −6.99, *p*_*males*_<0.01, Figs. 2A–2B). Regardings fish size, the *b* value of mature fish (2.80 ± 0.37 SE, *n* = 63, Fig. 2D) was higher than that of immature fish (2.62 ± 0.06 SE, *n* = 423, Fig. 2C). The immature fish showed negative allometry due to *b*<3 (*df* = 421, *t* =  − 6.18, *p* <  0.01, Fig. 2C), whereas the mature fish displayed isometry because of *b* ≈3 (*n* = 63, *df* = 61, *t* =  − 1.99, *p* = 0.05, Fig. 2D). Similar to size, the growth pattern of *P. gracilis* changed with the season variable as it showed negative allometry in the dry season (*b* = 2.51 ± 0.08, <3, *df* = 195, *t* =  − 6.25, *p* <  0.01, Fig. 2E) but isometry in the wet season (*b* = 2.87 ± 0.08, ≈3, *p* = 0.08, *t* =  − 1.79, Fig. 2F). Growth pattern of this mudskipper varied with site as it showed negative allometry at TV, ST and BL but isometry in CM. Indeed, *b* values of this fish at TV (2.79 ± 0.10 SE, *n* = 139, Fig. 3A), ST (2.11 ± 0.10 SE, *n* = 95, Fig. 3B) and BL (2.68 ± 0.09, *n* = 129, Fig. 3C) were <3 (*df*_*TV*_ = 137, *t*_*TV*_ = −2.05, *p*_*TV*_ = 0.04; *df*_*ST*_ = 93, *t*_*ST*_ = −8.83, *p*_*ST*_<0.1; *df*_*BL*_ = 127, *t*_*BL*_ = −3.43, *p*_*BL*_<0.01). By contrast, this value in CM (*b* = 2.96 ± 0.11, *n* = 123, Fig. 3D) was ≈3 (*df* = 121, *t* =  − 0.41, *p* = 0.69). The lowest value of *b*-value in ST could suggest that most of fish caught from ST belonged to immature fish.

The growth pattern of this fish was also changed with the month variable as it showed negative allometry to isometry to positive allometry. Spefically, as *b* value in February (2.39 ± 0.16 SE, *n* = 33), April (2.35 ± 0.27 SE, *n* = 34), May (2.56 ± 0.19SE, *n* = 46), June (2.43 ± 0.23SE, *n* = 37), August (2.07 ± 0.20 SE, *n* = 51) was signicantly less than 3 (*df*_April_ = 32, *t*_April_ = −2.42, *p*_April_ = 0.02; *df*_May_ = 44, *t*_May_ = −2.31, *p*_May_ = 0.03; *df*_June_ = 35, *t*_June_ = −2.50, *p*_June_ = 0.02; *df*_August_ = 49, *t*_August_ = −4.71, *p*_August_ < 0.01; *df*_February_ = 31, *t*_February_ = −3.80, *p*_February_ < 0.01), this fish showed negative allometry. Only in September, the species displayed positive allometry since its *b* value (*b* = 3.47 ±.01, *n* = 42) was >3 (*df* = 40, *t* = 3.47, *p* < 0.01). By contrast, this fish showed isometric growth as *b* value of this fish in the remaning months, *e.g.*, in January (2.67 ± 0.17, *n* = 35), March (2.84 ± 0.16, *n* = 49), July (2.49 ± 0.26, *n* = 43), October (3.03 ± 0.17, *n* = 43), November (2.93 ± 0.13, *n* = 38) and December (3.14 ± 0.25, *n* = 35), equivalented to 3 (*df*_July_ = 44, *t*_*J*__uly_ = −1.98, *p*_July_ = 0.03; *df*_October_ = 41, *t*_October_ = 0.18, *p*_October_ = 0.86; *df*_November_ = 38, *t*_November_ = −0.52, *p*_November_ = 0.60; *df*_December_ = 35, *t*_December_ = 0.56, *p*_December_ = 0.58; *df*_January_ = 33, *t*_January_ = −1.99, *p*_January_ = 0.05; *df*_March_ = 41, *t*_March_ =  − 1.00, *p*_March_ = 0.32) (Fig. 4).

### Condition factor

Condition factor (*CF*) of female *P. gracilis* (1.09 ± 0.02 SE, *n* = 236) was higher than that of males (0.96 ± 0.01, *n* = 250) (*n* = 486, *df* = 484, *t* = 5.94, *p* < 0.01, CI 95% = [0.12–0.05]). The *CF* of both sexes were significantly larger than 1 (*n*_females_ = 236, *df*_females_ = 235, *t*_females_ = 4.92, *p*_females_ < 0.01, CI 95%_females_ = [0.17 to −0.07]; *dfn*_males_ = 250, _males_ = 249, *t*males = −3.33, *p*_*males*_ = 0.01, CI 95%_males_ = [−0.01 to −0.06]). Likewise, the *CF* of immature fish group (1.01 ± 0.01 SE, *n* = 423) was significantly less than that of mature one (1.12 ± 0.03 SE, *n* = 63) (*df* = 484, *t* =  − 3.19, *p* < 0.01, CI 95% = [(−0.04)–(−0.17)]). The *CF* of immature fish was ≈1 (*n* = 423, *df* = 422, *t* = 0.90, *p* = 0.36, CI 95% = [0.03–(−0.01)]); this value of mature fish was >1 (*n* = 63, *df* = 61, *t* = 4.50, *p* <  0.01, CI 95% = [0.17–0.06]). The variation of *CF* was also found in season variable as this value in the dry season (0.99 ± 0.01 SE, *n* = 197) was lower than in the wet season (1.05 ± 0.02 SE, *n* = 289) (*df* = 484, *t* =  − 2.74, *p* < 0.01, CI 95% = [(−0.02)–(−0.10)]. In the dry season, the *CF* was ≈1 (*n* = 197, *df* = 196, *t* =  − 0.88, *p* = 0.38, CI 95% [0.15–(−0.03)]); however, in the wet season the *CF* increases and was >1 (*n* = 289, *df* = 288, *t* = 3.09, *p* <  0.01, CI 95% = [0.08–0.01]).

**Figure 5:**
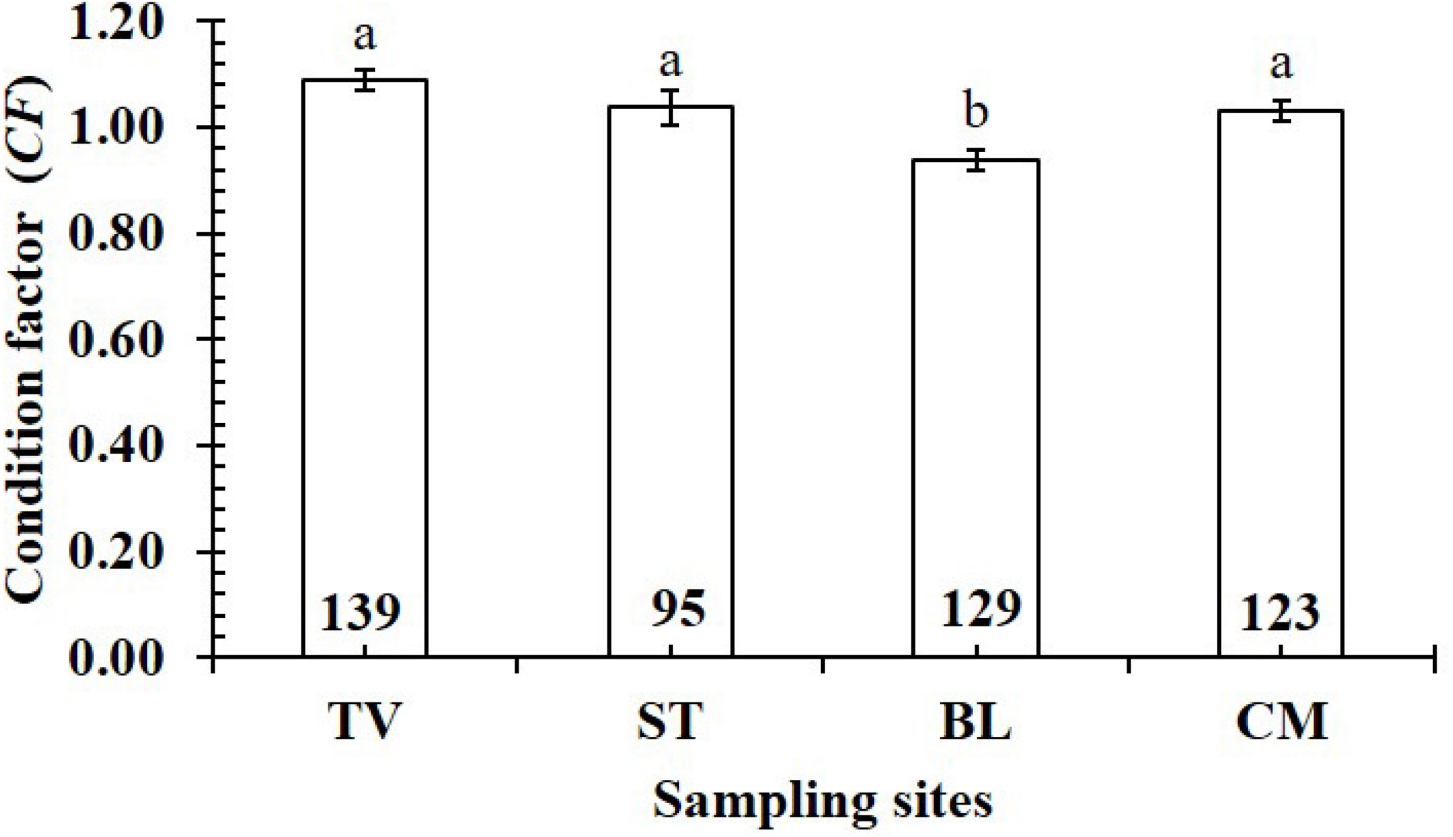
Variations of condition factor of * P. gracilis* by site. TV, Duyen Hai–Tra Vinh; ST, Tran De–Soc Trang; BL, Dong Hai–Bac Lieu; CM, Dam Doi–Ca Mau; the vertical bar is standard error of mean; a and b represent the significant difference; the number in each column is number of samples.

**Figure 6:**
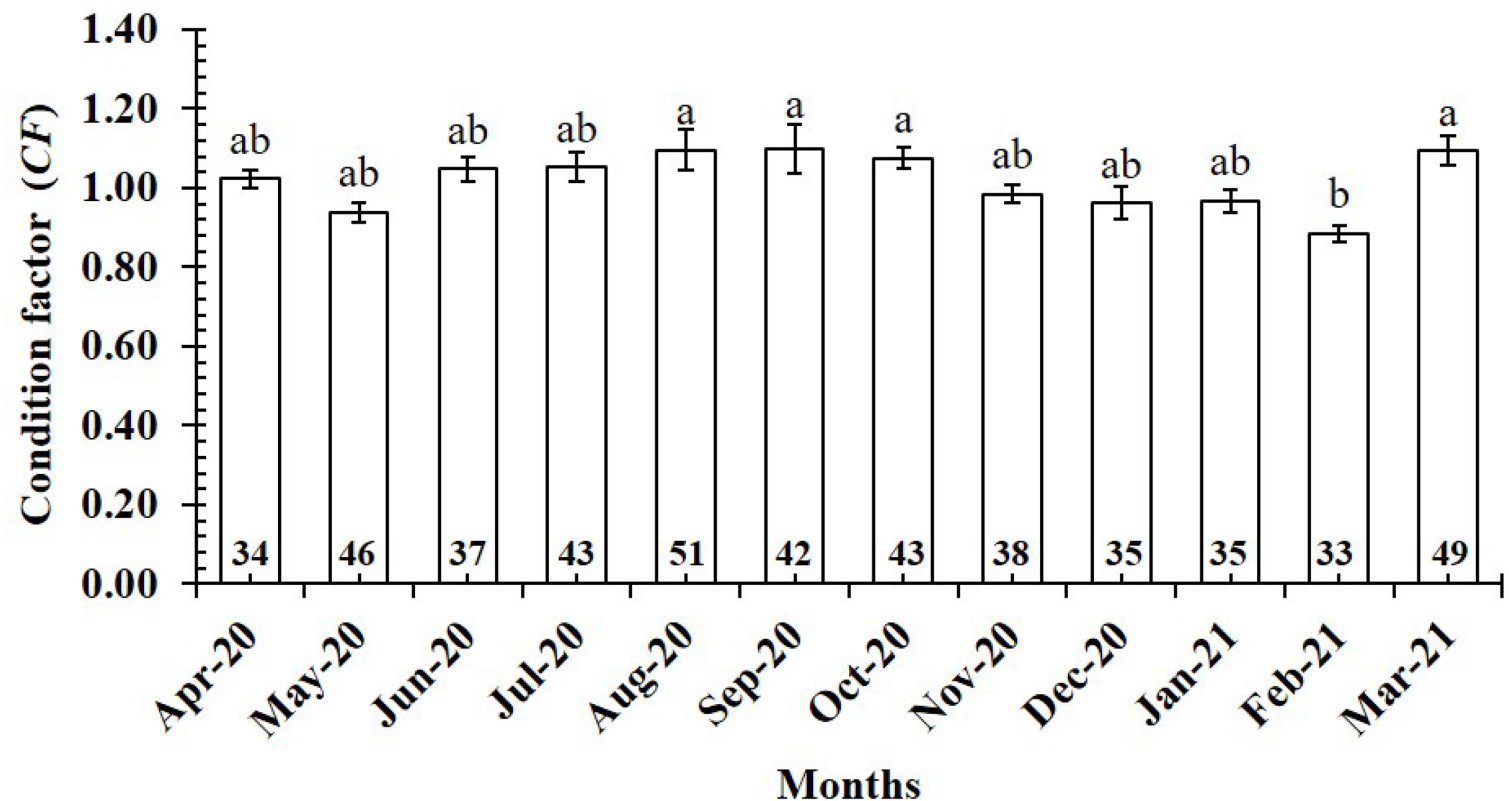
Variations of condition factor of * P. gracilis* by month. Vertical bar was standard error of mean; a and b represented the significant difference; number in each column was number of samples.

The *CF* value of *P. gracilis* varied with site (one-way ANOVA, *n* = 486, *df* = 3, *F*_2,3_ = 4.56, *p* <  0.01). This value at BL (0.94 ± 0.02 SE, *n* = 129) was significantly lower than that at the remaining 3 sites, comprising TV (1.09 ± 0.02 SE, *n* = 139), ST (1.04 ± 0.03 SE, *n* = 95) and CM (1.03 ± 0.02 SE, *n* = 123) (Fig. 5). At ST and CM, the *CF* of this fish was equivalent to 1 (*n*_*ST*_ = 95, *df*_*ST*_ = 94, *t*_*ST*_ = 1.19, *p*_*ST*_ = 2.35, CI 95%_*ST*_ = [0.09 to −0.02]; *n*_*CM*_ = 123, *df*_*CM*_ = 122, *t*_*CM*_ = 1.74, *p*_*CM*_ = 0.08, CI 95%_*CM*_ = 0.06 − (−0.01)). Meanwhile at BL, the *CF* was less than 1 (*n* = 129, *df* = 128, *t* =  − 3.09, *p* <  0.01, CI 95%= [−0.02 to −0.09]). and at TV this value was higher than 1 (*n* = 139, *df* = 138, *t* = 4.37, *p* <  0.01, CI 95% [0.12–0.04]). The *CF* of this fish fluctuated during the 12-month study (*n* = 486, *df* = 11, *F*_2,11_ = 3.43, *p* <  0.01), reaching the highest value in March and August-October (1.07 ± 0.04 SE to 1.10 ± 0.06 SE) and the lowest value in February (0.89 ± 0.02 SE, *n* = 33) (Fig. 6).

**Figure 7:**
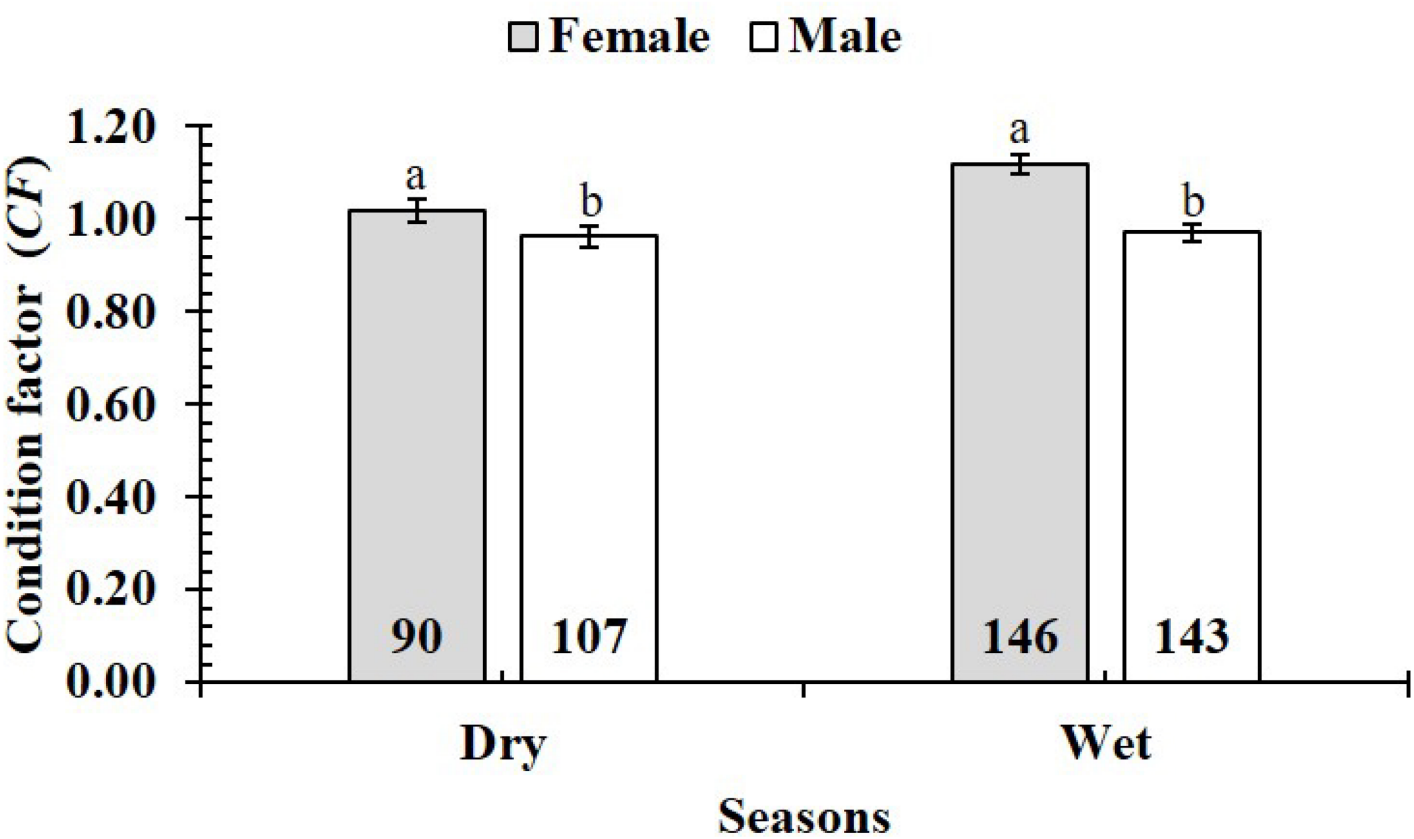
Variations of condition factor of* P. gracilis* by sex and seaon interaction. The vertical bar is standard error of mean; a and b represent the significant difference; the number in parentheses is number of samples.

**Figure 8:**
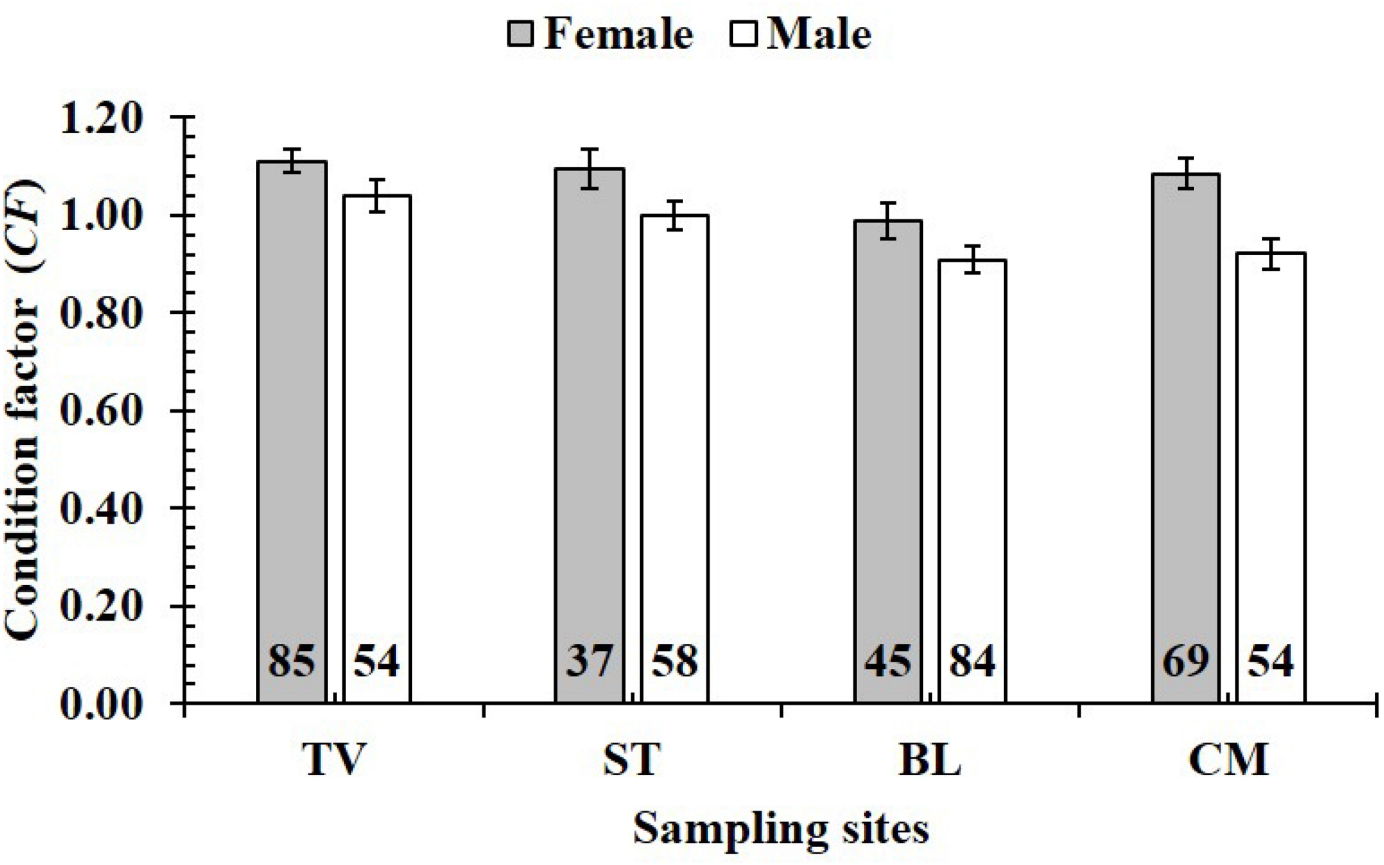
Variations of condition factor of * P. gracilis* by sex and site interaction. TV, Duyen Hai–Tra Vinh; ST, Tran De–Soc Trang; BL, Dong Hai–Bac Lieu; CM, Dam Doi–Ca Mau; vertical bar was standard error of mean; a and b represented the significant difference; number in parentheses was number of samples.

**Figure 9:**
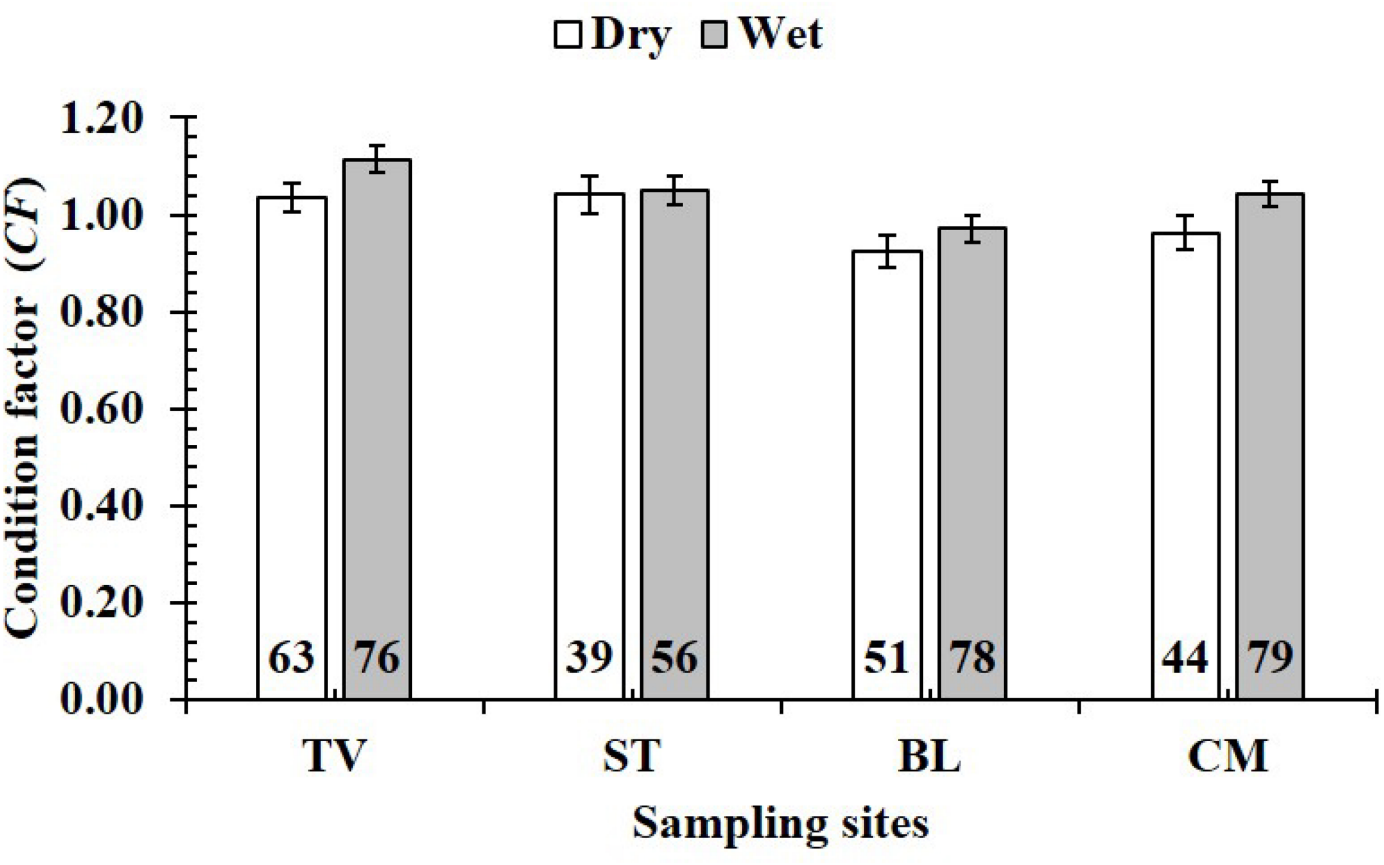
Variations of condition factor of * P. gracilis* by season and site interaction. TV, Duyen Hai–Tra Vinh; ST, Tran De–Soc Trang; BL, Dong Hai–Bac Lieu; CM, Dam Doi–Ca Mau; vertical bar was standard error of mean; a and b represented the significant difference; number in parentheses was number of samples.

The *CF* was also changed by the interaction of sex × season (GML, *n* = 486, *F*_2,3_ = 4.18, *p* = 0.04) (Fig. 7), but sex × site (*n* = 486, *F*_2,3_ = 0.91, *p* = 0.44) (Fig. 8) and season × site (*n* = 486, *F*_2,3_ = 0.31, *p* = 0.82) (Fig. 9). In overall, *CF* value of this mudskipper (1.05 ± 0.02, *n* = 495) was significantly higher than an ideal threshold value of 1 (*t* = 2.21, *n* = 486, *df* = 485, *p* = 0.03, CI 95% = [0.05–0.01]).

## Discussion

As high determination values (*r*^2^) of *LWRs*, fish weight in each sex, size, month, season, and site could be estimated from a given length, showing that fish weight could be obtained from fish length regardless of fish developmental stage. Similarly, positive relationships between *TL* and *W* were found in its congeners, *e.g.*, *P. barbarus* in Nigeria (Chukwu & Deekae, 2011), *P. argentilineatus* and *P. gracilis* in Indonesia (Taniwel & Leiwakabessy, 2020) and *P. modestus* in the northern of Vietnam (Tran, Nguyen & Ha, 2021b). Some gobies living in MD, *e.g.*, *Glossogobius sparsipapillus* (Dinh, 2015; Truong et al., 2021), *P. serperaster* (Dinh et al., 2016), *Butis butis* (Dinh, 2017a), *B. koilomatodon* (Lam & Dinh, 2021), *G. giuris* (Dinh & Ly, 2014; Phan et al., 2021b) and *G. areus* (Phan et al., 2021a) also displayed a positive relationship between *TL* and *W*.

Since *b* value was less than 3, *P. gracilis* displayed negative allometry, showing that most fish collected corresponded to the immature stage found in its congeners, *e.g.*, *P. barbarus* in Nigeria (*b* = 2.73) (King & Udo, 1998). A previous study on the genus *Periophthalmus* in Indonesia showed that both *P. argentilineatus* and *P. gracilis* displayed negative allometry (*b*<3) (Taniwel & Leiwakabessy, 2020). As *b* <3, the negative allometric pattern was observed in some gobies living in MD, *e.g.*, *G. aureus* (*b* = 2.71) (Dinh, 2014b; Phan et al., 2021b) and *B. koilomatodon* (*b* = 2.66) (Lam & Dinh, 2021) and *G. sparsipapillus* (*b* = 2.68) (Truong et al., 2021). Meanwhile, other gobies living in MD showed isometric growth, *e.g.*, *B. boddarti* (Dinh, 2014a), *P. serperaster* (Dinh et al., 2016), *T. vagina* (Dinh, 2016b), *P. schlosseri* (Dinh, 2016c) and *G. giuris* (Dinh & Ly, 2014; Phan et al., 2021b) because of *b* ≈3. Similar to *P. gracilis*, other congeners of *P. gracilis*, *e.g.*, *P. modestus* distributing in the Red River Delta (RD), north of Vietnam, showed positive allometry (*b*>3) (Tran, Nguyen & Ha, 2021b). Likewise, the positive allometric growth pattern was also found in *P. chrysospilos* occurring in Malaysia (Abdullah & Zain, 2019) and *P. kalolo* and *P. malaccensis* in Indonesia (Taniwel & Leiwakabessy, 2020). Some other fish species living in MD, *e.g.*, *S. pleurostigma* displayed positive allometry (*b* > 3) (Dinh, 2017b). The similarities and differences in growth patterns among these gobies indicated fish growth type was specific-species and regulated to by environment.

The difference in ovarian and testicular weights did not regulate fish growth patterns as male and female *P. gracilis* showed negative allometry. A similar growth pattern in the two sexes was found in its congeners in Nigeria, *e.g.*, *P. barbarus* also showed (King & Udo, 1998), but not in *P. modestus* living in the RD (Tran, Nguyen & Ha, 2021b). As immature groups showed negative allometry, but mature groups showed isometric growth regarding the fish size. It showed that the growth pattern of *P. gracilis* was impacted by fish size, which was found in *P. barbarus* in Nigeria (King & Udo, 1998) but not in *P. modestus* in RD (Tran, Nguyen & Ha, 2021b). Similar to fish size, the growth pattern of *P. gracilis* in the dry season was different from in the wet season, seeming that the difference in precipitation between these two seasons affected fish growth type, which was found in its congener –*P. modestus* in RD (Tran, Nguyen & Ha, 2021b). The spatial variation in the growth type of *P. gracilis* could be related to differences in abiotic factors among these sites (Dinh et al., 2021a), which was also found in *P. waltoni* in Nigeria (Sarafraz et al., 2012) and *P. modestus* in RD (Tran, Nguyen & Ha, 2021b). Like *P. gracilis*, the variation in growth pattern among months was found in some gobies living in and out of MD, *e.g.*, *P. barbarus* (King & Udo, 1998), *P. waltoni* (Sarafraz et al., 2012), *G. giuris* (Dinh & Ly, 2014; Phan et al., 2021b), *B. boddarti* (Dinh, 2014a), *P. serperaster* (Dinh et al., 2016), *T. vagina* (Dinh, 2016b), *P. schlosseri* (Dinh, 2016c) and *P. modestus* (Tran, Nguyen & Ha, 2021b).

The *CF* of *P. gracilis* was affected by gender and size, indicating that fish body condition could relate to fish developmental stages. In Nigeria, *CF* its congener, *P. barbarus*, did not show sexual changes in *CF* (King & Udo, 1998; Chukwu & Deekae, 2011). However, *P. modestus*, another congener in RD, showed a sexual change in *CF* as this value was high in females towards the end of gonadal maturation, which was found in *P. serperaster* living in MD (Dinh et al., 2016). Different from *P. gracilis*, *CF* of some gobies in MD, *e.g.*, *P. serperaster* (Dinh et al., 2016), *P. schlosseri* (Dinh, 2016c), *T. vagina* (Dinh, 2016b), and *G. giuris* (Phan et al., 2021b) did not vary with fish size. The wet season was observed preferably for *P. gracilis* as *CF* in the wet season was higher than in the dry season, whereas a reverse case was found *P. modestus* in RD due to a lower *CF* in the wet season compared to the dry season (Tran, Nguyen & Ha, 2021b). Different from *P. gracilis*, a similar in *CF* between dry and wet seasons were also found in co-occurring gobiid species such as *P. elongatus* (Tran, 2008), *P. serperaster* (Dinh et al., 2016), *T. vagina* (Dinh, 2016b), *G. giuris* (Phan et al., 2021b) and *B. koilomatodon* (Lam & Dinh, 2021). Fish body condition factors could be regulated by the variation in biotic factors between four sites due to the spatial variation in *CF*. This assumption was also found in co-occurring goby *B. koilomatodon* (Lam & Dinh, 2021) but not in *P. modestus* in RD (Tran, Nguyen & Ha, 2021b). Although *P. gracilis* showed spatiotemporal variation in *CF*, the research sites contributed favourable environmental conditions as its *CF* was higher than the threshold of 1. Likewise, its congeners living out of MD was also adapted well to their habitats due to higher *CF*, *e.g.*, *P. barbarus* (King & Udo, 1998), *P. chrysospilos* (Abdullah & Zain, 2019; Dinh et al., In Press), *P. modestus* (Tran, Nguyen & Ha, 2021b) and *P. variabilis* (Dinh et al., 2022). This assumption was also found in some other fish species in MD, such as *P. elongatus* (Tran, 2008), *P. serperaster* (Dinh et al., 2016), *T. vagina* (Dinh, 2016b), *P. schlosseri* (Dinh, 2016c) and *G. aureus* (Dinh, 2019) and *G. giuris* (Phan et al., 2021b).

## Conclusions

As the slope value obtained from *LWR* was less than 3, *P. gracilis* displayed negative allometry for both sexes, showing that most individual fish was caught in the immature stage. The growth pattern did not show sexual changes but intraspecific and spatiotemporal variations. The *CF* was regulated by gender, fish size and season, sites and month, and this value of this species was higher than 1, showing it adapted well to the environment. The fish length at first capture should be increased in order to conserve this species.

##  Supplemental Information

10.7717/peerj.13060/supp-1 Supplemental Information 1 Raw data

## Additional Information and Declarations

### Data Availability

The following information was supplied regarding data availability: The raw data are available in the Supplementary Files.
